# Highly-efficient synthesis of biogenic selenium nanoparticles by *Bacillus paramycoides* and their antibacterial and antioxidant activities

**DOI:** 10.3389/fbioe.2023.1227619

**Published:** 2023-08-01

**Authors:** Pei Liu, Haiyu Long, Han Cheng, Mengdi Liang, Zhengwei Liu, Zhenlian Han, Zhen Guo, Hao Shi, Min Sun, Shuai He

**Affiliations:** ^1^ Faculty of Life Science and Food Engineering, Huaiyin Institute of Technology, Huaian, China; ^2^ Jiangsu Provincial Key Construction Laboratory of Probiotics Preparation, Huaiyin Institute of Technology, Huaian, China; ^3^ Jiangsu Key Laboratory for Eco-Agricultural Biotechnology around Hongze Lake, School of Life Science, Huaiyin Normal University, Huaian, China; ^4^ Huai’an Municipal Center for Disease Control and Prevention, Huaian, China

**Keywords:** selenium nanoparticles, *Bacillus paramycoides*, antibacterial activity, antioxidant activity, green synthesis

## Abstract

**Introduction:** *Bacillus* species are known for their ability to produce nanoparticles with various potential applications.

**Methods:** In this study, we present a facile approach for the green synthesis of selenium nanoparticles (Se NPs) using the biogenic selenate-reducing bacterium *Bacillus paramycoides* 24522. We optimized the growth conditions and sodium selenite reduction efficiency (SSRE) of *B. paramycoides* 24522 using a response surface approach.

**Results:** Se NPs were synthesized by reducing selenite ions with *B. paramycoides* 24522 at 37 °C, pH 6, and 140 r/min, resulting in stable red-colored Se NPs and maximal SSRE (99.12%). The synthesized Se NPs demonstrated lethality against *Staphylococcus aureus* and *Escherichia coli* with MICs of 400 and 600 μg/mL, and MBCs of 600 and 800 μg/mL, respectively, indicating the potential of Se NPs as antibacterial agents. Furthermore, the Se NPs showed promising antioxidant capabilities through scavenging DPPH radicals and reducing power.

**Discussion:** This study highlights the environmentally friendly production of Se NPs using *B. paramycoides* 24522 and their possible applications in addressing selenium pollution, as well as in the fields of environment and biotechnology.

## 1 Introduction

**FIGURE 1:**
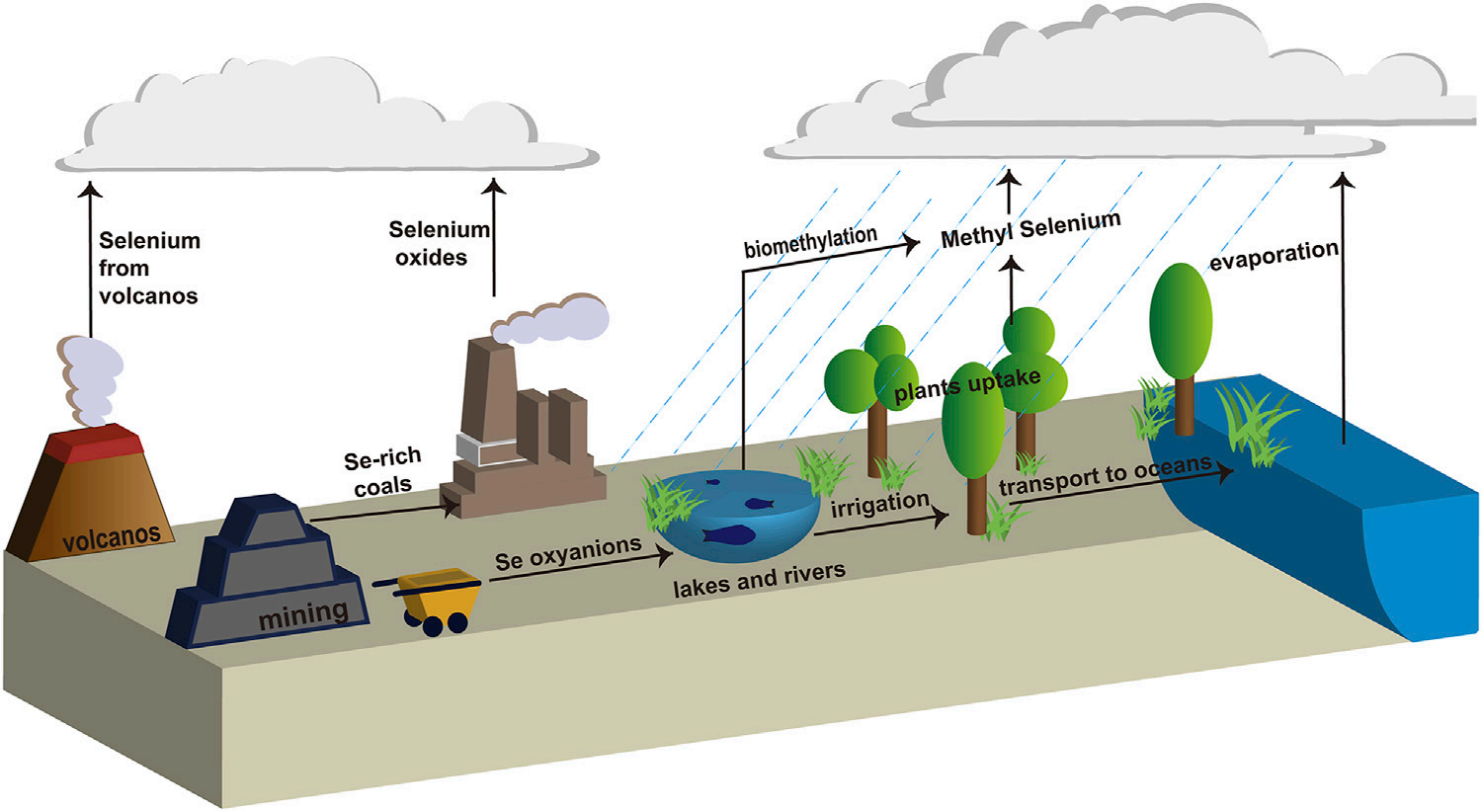
Biogeochemical cycle of selenium.

Selenium (Se) plays a vital role in the construction of plants and biological systems at lower concentrations (Al-Hagar et al., 2021). As shown in Figure 1 Se is present in different oxidation states, such as soluble Se oxyanions (SeO_4_^2−^ and SeO_3_^2−^), which are released by geologic and anthropogenic sources (volcanos and mining) into the environment, such as lakes and rivers (Song et al., 2017). These Se oxyanions can easily be assimilated and bioaccumulated by fish, shrimp, or some other aquatic animals from contaminated rivers, lakes, or oceans, adversely affecting the health of human beings (Shi et al., 2021). Therefore, Se contamination of water resources has become an increasingly severe health risk issue (Jadhav et al., 2022). Due to the toxicity of Se oxyanions, the maximum acceptable content in drinking water assigned by the World Health Organization (WHO) is 40 μg/L and 10 μg/L in the Bureau of Indian Standards (BIS) guidelines (Sonkeshariya et al., 2020). Additionally, the Ministry of Environment and Climate Change Strategy in Canada assigned a policy for background Se concentrations of 2 μg/L in the water columns and sediment (English et al., 2022). In this regard, eliminating and/or reducing the adverse effects caused by high concentrations of Se oxyanions has become increasingly imminent.

Selenium nanoparticles (Se NPs), however, exhibit low or no cytotoxicity when compared with Se oxyanions (Xu et al., 2018; He et al., 2021). Recently, Se NPs have received widespread attention because of their unique properties, such as antioxidant (Azimi et al., 2021; Brar et al., 2022), photocatalytic (Velayati et al., 2022), antibacterial and anticancer activities (Han et al., 2020; Rana, 2021). Therefore, the development of green, efficient and sustainable biotechnological synthetic routes for transforming toxic Se oxyanions into nontoxic Se NPs is of great significance.

There are numerous techniques available for the production of Se NPs, including chemical reduction, physical processes, and biological approaches. Nonetheless, these methods are associated with drawbacks such as high expenses, limited safety, significant toxicity, and environmental contamination (Shreyash et al., 2021). Microbial cells possess a distinctive metabolic pathway that allows them to generate unique metabolites with special characteristics, including enzymes and bioactive substances. As living organisms, they offer great potential for exploring new avenues in reducing the soluble toxic selenium oxides to the insoluble hypotoxic selenium element (Se0), which in turn forms Se NPs (Zhang et al., 2018). *Bacillus* strains, such as *Bacillus mycoides* SeITE01 (Lampis et al., 2014), *Bacillus subtilis* 168 (Jia et al., 2022), and *Bacillus niabensis* OAB2 (Al-Hagar et al., 2021), have shown promise as nano-factories capable of producing a range of Se NPs, when culturing these bacteria in media containing nutrients and the precursor selenite. The reaction mixture changes from colorless to reddish color, generally indicating successful synthesis of SeNPs. Some Gram-negative bacteria, such as *Stenotrophomonas maltophilia* SeITE02 (Wadhwani et al., 2016), *Shewanella* sp*.* 023S (Staicu et al., 2022), *Pseudomonas aeruginosa* ATCC 27853 (Kora and Rastogi, 2016), *Escherichia coli* ATCC 35218 (Kora and Rastogi, 2017), *Enterobacter cloacae* Z0206 (Song et al., 2017), and *Lactobacillus casei* ATCC 393 (Qiao et al., 2020) have also been reported to synthesize Se NPs.

Among the microorganisms that have been reported, *B. paramycoides* is a valuable microbe that can efficiently degrade organic matter and eliminate pollutants. This bacterium possesses excellent reduction abilities and can reduce selected metal ions, transforming them into nanoparticles (Yadav et al., 2023). In a study by (Dharmaraj et al., 2021), zinc oxide nanoparticles (ZnO NPs) were synthesized using the cell-free supernatant of *B. paramycoides*. Additionally, the culture supernatant of this bacterium was utilized to produce silver oxide nanoparticles (Ag_2_O NPs), which were evaluated for their potential antibiofilm activity and cytotoxic effects. These findings suggest that *B. paramycoides* could be a promising organism for the synthesis of nanoparticles with potential environmental remediation and waste management applications. Its potential should be further explored to unlock its full benefits for society.

In this study, an aquatic-derived *B. paramycoides* 24522 with remarkable reduction efficiency of selenite was isolated from selenium-rich aquaculture pond sediment in China. Factors affecting *B. paramycoides* 24522 growth and sodium selenite reduction efficiency (SSRE) were optimized using response surface methodology. The Se NPs were characterized using dynamic light scattering (DLS), X-ray diffraction (XRD), Fourier transform infrared (FT-IR) spectroscopy, and scanning electron microscopy-energy dispersive spectrometry (SEM-EDS). The antimicrobial and antioxidant capabilities of Se NPs were also evaluated.

## 2 Materials and methods

### 2.1 Pond sediment samples and chemicals

Sediment samples were collected from the Hongze Lake aquaculture pond (33°02′N, 118°28′E) in Huai’an, China. To generate selenium-rich aquaculture pond sediment, sodium selenite (Na_2_SeO_3_) powder was added to the sediment and cultivated for 30 days, sprayed with water and stirred thoroughly 5–6 times every day. Na_2_SeO_3_ and all the other analytical grade chemicals were purchased from Sinopharm Chemical Reagent Co., Ltd. (Shanghai, China).

### 2.2 Selenate-reducing microorganism isolation and identification

One gram of selenium-rich aquaculture pond sediment was dispensed into 100 mL of LB medium (5 g/L peptone, 3 g/L beef extract, 5 g/L NaCl) and incubated at 37°C with shaking at 200 rpm for 1 week. After that, 1 mL of the upper liquid was added into 9 mL sterilized deionized water, and this was a 10^−1^ dilution. Following a similar procedure, we prepared 10^−2^–10^−6^ dilutions. To screen Se NP-producing microorganisms, 5 mM Na_2_SeO_3_ was first added to solid LB plates, and 0.2 mL of each dilution was then added, spread and placed at 37 °C for 1–7 days. The isolated microorganisms were purified by streaking on a plate many times. Single colonies were picked out and stored at 4°C for further experiments.

For screening efficient selenate-reducing microbes, 0.1 mL of the single isolated colonies were inoculated in 100 mL LB liquid medium with 5 mM Na_2_SeO_3_ and cultured at 37°C for 24 h. The fermentation broth turned reddish in 24 h, indicating that the corresponding microbes possess a relatively high conversion ability of Na_2_SeO_3_. The most efficient selenate-reducing isolate, 1805, was identified based on its physiological/biochemical characteristics with the help of 16S rDNA sequencing. PCR products of isolate 1805 were viewed on an agarose gel (1% w/v), sequenced, and BLAST searched through the National Center for Biotechnology Information (NCBI) database (https://www.ncbi.nlm.nih.gov/). The phylogenetic tree of strain 1805 was plotted using MEGA 11 software (Tamura et al., 2021).

### 2.3 Optimization of *B. paramycoides* 24522 growth conditions

The growth curve of *B. paramycoides* 24522 was plotted by culturing it in LB broth at 37°C and 200 rpm for 48 h. The effects of temperature (20, 25, 30, 37, 40, 42°C), pH (4, 5, 6, 7, 8, 9, 10, 11) and rotation speed (0, 50, 100, 150, 200, 250, 300, 350 rpm) on the growth of *B. paramycoides* 24522 were determined.

### 2.4 Optimization of SSRE conditions of *B. paramycoides* 24522

#### 2.4.1 Determination of sodium selenite concentration and SSRE

The sodium selenite concentration was determined by the Na_2_S chromogenic method following the published protocols of (Biswas et al., 2011). In brief, a standard curve (Supplementary Figure S1) of elemental selenium was first plotted by accurately adding 1, 2, 4, 6, 8, 10, 12, 14, 16, 18, and 20 mg of elemental selenium powder into 30 mL of sodium sulfide nonahydrate solution (1 M) and mixing thoroughly. The absorbances were measured at a wavelength of 500 nm (OD_500_), taking sodium sulfide nonahydrate solution without selenium powder as a control.

After that, the content of synthetic elemental selenium in the culture medium was determined by inoculating 1% of *B. paramycoides* 24522 into LB medium with 2 mM Na_2_SeO_3_, which was prepared as a 1 M stock solution and filter sterilized. The cells were cultured at 37°C and 200 rpm for 24 h. The mixture was centrifuged at 5,000×g and 4°C for 20 min, the supernatant was discarded, and the sample was washed and centrifuged three times with 1 M NaCl (5,000×g, 20 min, resuspended in 30 mL of 1 M sodium sulfide nonahydrate solution for 30 min. The sample was centrifuged again at 10,000×g for 30 min. The absorbance at OD_500_ of the supernatant liquid was measured using *B. paramycoides* 24522 cultures for 24 h without adding Na_2_SeO_3_ and under the same operation as the control. The content of synthetic elemental selenium was calculated by a standard curve.

SSRE was calculated as follows:

$$SSRE=\frac{Content\;of\;synthetic\;elemental\;selenium}{Content\;of\;elemental\;selenium\;in\;added\;{Na}_{2}SeO_{3}}\times 100\%$$

#### 2.4.2 Effect of temperature, pH and rotation speed on the SSRE of *B. paramycoides* 24522

The effects of temperature (20, 25, 30, 35, 37, 40, 42, 45°C), pH (2, 4, 6, 8, 10, 12), and rotation speed (0, 50, 100, 150, 200, 250, 300, 350 rpm) on SSRE of *B. paramycoides* 24522 were studied in the presence of 2 mM Na_2_SeO_3_. SSRE under different conditions was measured and calculated as described in Section 2.4.1.

#### 2.4.3 Optimization of SSRE using response surface methodology

Response surface methodology was used to optimize the conditions for SSRE of *B. paramycoides* 24522. The experiment was conducted using Box-Behnken design (BBD) with Design-Expert version 12 software. The pH (A), temperature (B), and rotation speed (C) were detected as independent variables according to the results of the above single-factor experiments. SSRE was used as a response value. The factors and levels of these independent variables are presented in Supplementary Table S2. BBD was applied to obtain the second-order response surface.

### 2.5 Localization of Se NPs synthesized by *B. paramycoides* 24522

To determine the location and distribution of the biosynthesized Se NPs, *B. paramycoides* 24522 was inoculated into LB medium supplemented with 2 mM Na_2_SeO_3_. A negative control was set up with the isolate cultured in only LB medium. After incubation at 37°C for 24 h, the bacterial cultures were harvested through gentle centrifugation (5000×g for 10 min at 4°C). The strain was then subjected to cell fragmentation for the extraction of proteins and polysaccharides from each site. To begin, the supernatant and precipitate were collected. The supernatant contained exocytosis protein (ECP), while the precipitate was washed using 1×PBS buffer (pH7) and resuspended via centrifugation. Next, we centrifuged the supernatant at 10,000×g for 30 min to obtain periplasmic space protein (PSP). The precipitate from the previous step was dissolved in 1×PBS buffer (pH7) and treated with Dnase Ⅰ (125 mg/mL), which enzymatically cleaves phosphodiester bonds between DNA bases. Ultrasound was then used to further break up the sample, which was then centrifuged at 5000 *g* for 10 min to discard the precipitate and collect the supernatant, containing cytoplasmic protein (CPP). This precipitate was dissolved in 1×PBS buffer (pH7) to obtain cell membrane and cell wall protein (MWP). To obtain intracellular polysaccharides (IPS), 50 mL of ECP was filtered through a 0.22 um membrane. The resulting sample was then treated with an equal volume of ice ethanol and refrigerated overnight at −20°C. Finally, we centrifuged the sample at 12,000×g for 30 min at 4°C to obtain IPS.The isolated proteins and polysaccharides were incubated with sodium selenite, and changes in color activity were observed in EP tubules. All samples were kept on ice or at 4°C during the operation to prevent damage from external factors.

### 2.6 Recovery and characterization of the biosynthesized Se NPs

To purify Se NPs from the culture broth, a 24 h culture of *B. paramycoides* 24522 was grown in LB medium containing 2 mM Na_2_SeO_3_. The culture was then collected by centrifugation at 5000 *g* for 20 min and washed three times with 0.1 M phosphate buffered saline (PBS). The resulting pellets were subjected to an ultrasonication treatment using 500 W for 50 min of 2 s of sonication with 8 s of rest in between. The Se NPs were then harvested by centrifugation at 11,000×g for 20 min, following the method outlined in (Huang et al., 2021). This approach was successful in recovering Se NPs from the *B. paramycoides* 24522 culture, providing a potential avenue for further study of Se NPs and their applications.

To provide additional details on the properties of Se NPs, UV-Vis spectroscopy (MD SpectraMax Plus384, United States), DLS (Brookhaven Instruments NanoBrook 90Plus Zeta with Particle Solutions Ver. 3.5), XRD (Bruker, United States), FT-IR spectroscopy, and SEM-EDS were carried out as described in our previous work (Liu et al., 2022; Nie et al., 2022).

### 2.7 Antibacterial properties of Se NPs

In this study, the antibacterial properties of the as-prepared Se NPs at a concentration of 1 mg/mL were evaluated using the well diffusion method (Liu et al., 2022). *Staphylococcus aureus* ATCC 29213 and *Escherichia coli* ATCC 25922 were subcultured on nutrient agar and evenly coated on each plate. Then, 8 mm diameter wells containing the prepared Se NPs were added to the nutrient agar, along with controls of *B. paramycoides* 24522 culture (without Na_2_SeO_3_, 200 μL) and Na_2_SeO_3_ (200 μL, 2 mM). The plates were then incubated at 37°C for 24 h, after which the size of the inhibition zone was measured.

The minimum inhibitory concentration (MIC) and minimum bactericidal concentration (MBC) values of the biosynthesized Se NPs were determined according to a protocol published in our previous work (Nie et al., 2022). Different concentrations of Se NPs ranging from 100 to 1,000 μg/mL were added to the primary bacterial culture and incubated at 180 rpm and 37°C for 24 h. Bacterial growth was monitored using spectrophotometry at a wavelength of 600 nm (OD). The bacterial inhibition rate was determined using the formula below:

$$Bacterial\;inhibition\;rate\;\left(\%\right)=\frac{{OD}_{control}-{OD}_{sample}}{{OD}_{control}}\times 100$$

The MIC was determined as the concentration that hindered the normal growth of the test pathogens, and the MBC was defined as the concentration that completely suppressed the normal growth of the test pathogens.

### 2.8 Antioxidant activity of Se NPs

#### 2.8.1 DPPH free radical scavenging activity

The abilities of Se NPs, butyl hydroxy anisole (BHA), and butylated hydroxytoluene (BHT) as scavengers of 1,1-diphenyl-2-picrylhydrazyl (DPPH) radicals were evaluated using spectrophotometric techniques (Liu et al., 2022). A solution containing 6 mL of DPPH at a concentration of 0.06 mM was mixed with Se NPs (100 μg/mL) and then incubated in darkness for 30 min. Absorbance was measured at 517 nm against a blank. The scavenging capacity of DPPH was calculated as follows:

$$Scavenging\;capability\;\left(\%\right)=\frac{\Delta A_{517}\;of\;control-\Delta A_{517}\;of\;sample}{\Delta A_{517}\;of\;control}\times 100$$

#### 2.8.2 Reducing power

To evaluate the reducing power of the as-prepared Se NPs, we modified the method proposed by Mao (Mao et al., 2013). We prepared reaction mixtures by adding varying amounts of Se NPs (5, 10, 20, 30, 40, and 50 μg) into 2.5 mL of phosphate buffer (0.2 mol/L, pH 6.6) and 2.5 mL of potassium ferricyanide (1%). To start the experiment, the mixture was incubated in a water bath at 50°C for 20 min. Afterwards, 2.5 mL of 10% trichloroacetic acid (TCA) were added to each solution and then centrifuged at 1,000 *g* for 10 min. After separation, 2.5 mL of the supernatant was mixed with 2.5 mL of distilled water and 0.5 mL of FeCl_3_ (0.1%). The mixture was then allowed to react for 10 min, and the absorbance was measured at 700 nm. As control experiments, 0.1 g/L BHA and 0.1 g/L BHT were used.

### 2.9 Data analysis

The data were analyzed using Origin Pro 9 software and reported as the mean ± SD. To determine statistical significance (*p* < 0.05), one-way ANOVA was performed on the experimental results.

## 3 Results

### 3.1 Isolation, identification and characterization of the most efficient selenate-reducing microbe

**FIGURE 2:**
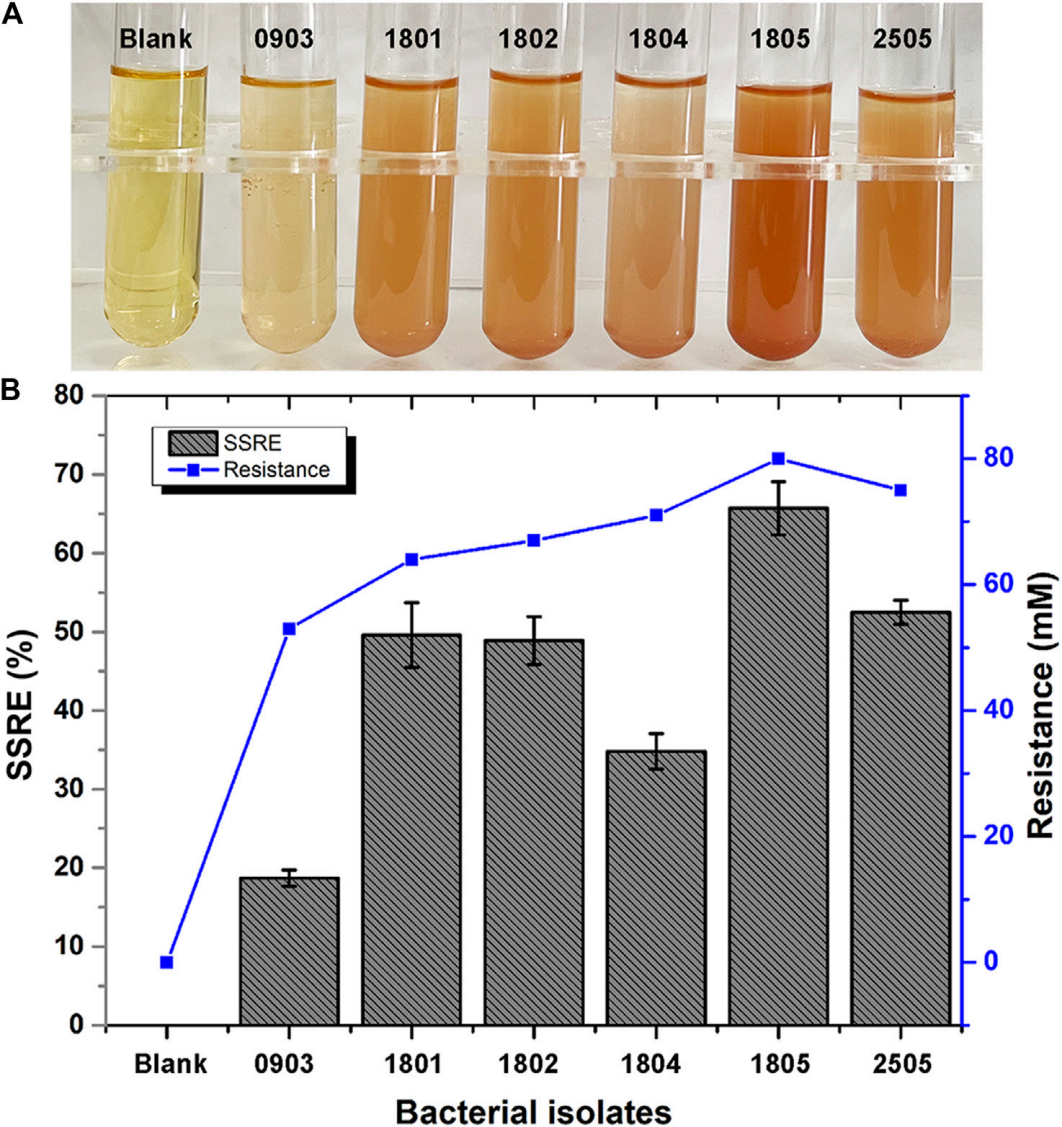
**(A)** Color changes during Se NPs biosynthesis (Blank: LB broth before synthesis, 0903, 1801, 1802, 1804, 1805, 2505: After synthesis with isolates 0903, 1801, 1802, 1804, 1805 and 2505). **(B)** Sodium selenite reduction efficiency (SSRE) of 6 strains after 24 h.

In this study, we investigated 162 isolates capable of converting Na_2_SeO_3_ into Se NPs within 48 h. These isolates were obtained from LB agar plates supplemented with 5 mM Na_2_SeO_3_. Six bacterial isolates (0903, 1801, 1802, 1804, 1805 and 2505) that could synthesize SeNPs within 24 h were further isolated from LB liquid medium with different concentrations of Na_2_SeO_3._ Of these strains, isolate 1805 exhibited the highest sodium selenite reduction efficiency (SSRE) and the greatest resistance to Na_2_SeO_3_ in the isolation medium (Figure 2). The bacteria capable of reducing Na_2_SeO_3_ appeared as reddish bacterial fluid in the culture medium, indicating the accumulation of Se NPs (Al-Hagar et al., 2021).

**FIGURE 3:**
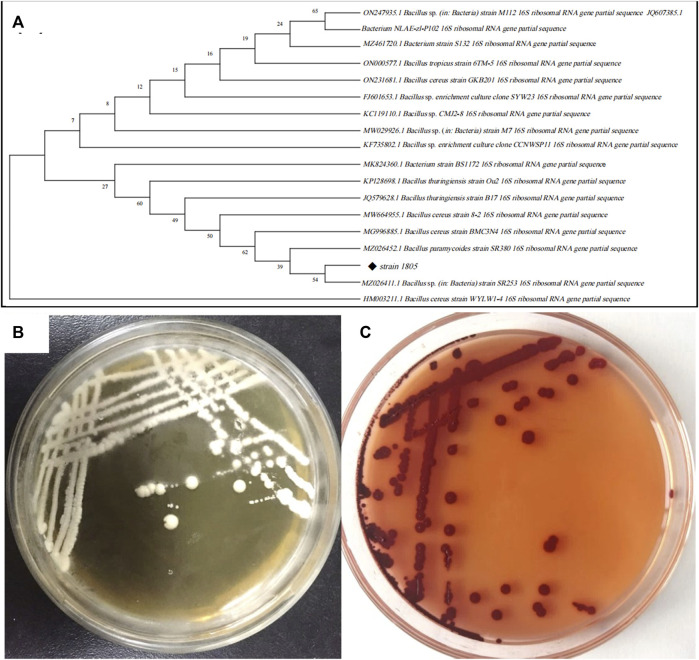
**(A)** Phylogenetic tree of strain 1805 and its related sequences from the NCBI database, **(B)** Growth of strain 1805 in solid LB medium, **(C)** Growth of strain 1805 in the presence of 5 mmol/L sodium selenite.

As shown in Figures 3B, C, isolate 1805 was milky white, opaque, viscous, and slightly protruding on the LB plate without Na_2_SeO_3_ but changed to dark red with supplementation of 5 mM Na_2_SeO_3_. Physiological and biochemical results indicated that strain 1805 was a Gram-positive bacterium that had contact enzyme activity to liquefy gelatin and peptonize milk and could also reduce nitrate and hydrolyze starch (Supplementary Table S1). The pH and temperature for isolate 1805 growth were 4–10 and 20°C–45°C, respectively, (Supplementary Table S1).

The best match model species of the 16S rDNA gene sequences to the isolate 1805 in NCBI GenBank are listed in Figure 3A, which revealed that this strain belonged to the genus *Bacillus* and had the highest similarities to the same species *B. paramycoides*, which scored 99.79%. Phylogenetic analysis using the neighbor-joining method (Abol-Fotouh et al., 2021) indicated that strain 1805 fell in the same clusters as *B. paramycoides* (MZ026452.1) (Figure 3A).

According to the appearance of the isolated colony, physiological/biochemical and sequencing results, the isolated strain was identified as *B. paramycoides*. This strain was preserved in the China General Microbiological Culture Collection Center (CGMCC, NO.24522). Therefore, this isolate was named *B. paramycoides* 24522.

### 3.2 Optimization of growth and SSRE of *B. paramycoides* 24522

The growth state of the inoculated bacteria is crucial for Se NPs biosynthesis. Therefore, we first studied the growth profile of *B. paramycoides* 24522 and observed that it grew rapidly from 4 h, entered a stable phase at 18 h, and declined after 24 h (Supplementary Figure S2). Therefore, it is necessary to select the optimal growth period for activation before 24 h, which provides a preliminary basis for optimizing the growth and SSRE conditions of *B. paramycoides* 24522.

#### 3.2.1 Growth optimization

**FIGURE 4:**
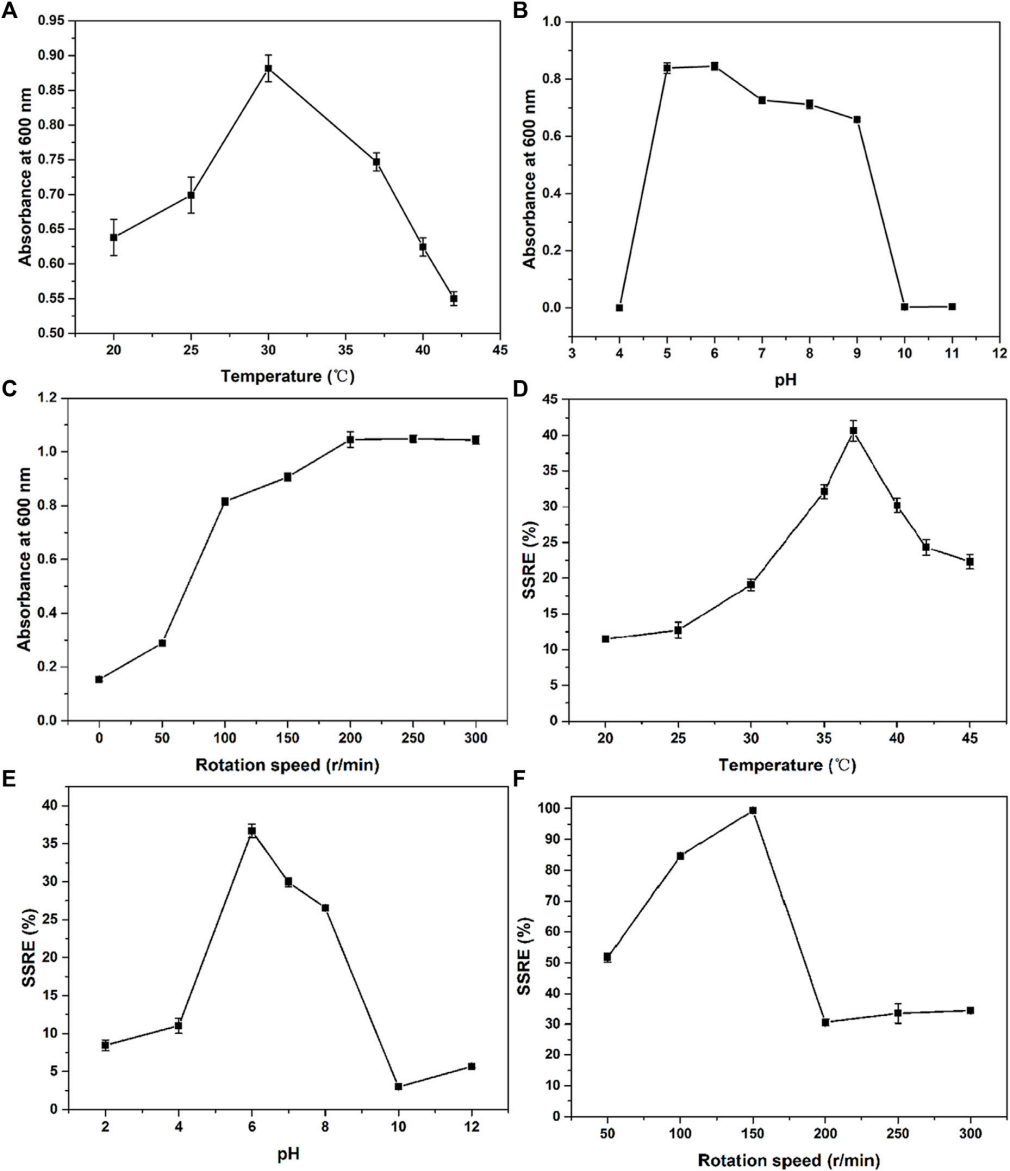
Effect of temperature **(A)**, pH **(B)** and rotation speed **(C)** on the growth and SSRE **(D–F)** of *B. paramycoides* 24522.

Optimum growth and fermentation conditions, such as temperature, pH, and rotation speed, of *B. paramycoides* 24522 for production as a biofactory are required but have not been found in the current literature. In this study, the effects of temperature (20, 25, 30, 35, 37, 40, 42, and 45°C), pH (2, 4, 6, 8, 10, and 12), and rotation speed (0, 50, 100, 150, 200, 250, 300, and 350 rpm) on the SSRE of *B. paramycoides* 24522 were studied in the presence of 2 mM Na_2_SeO_3_. As shown in Figures 4A–C, *B. paramycoides* 24522 grew best at 30°C, pH 6 and 200 rpm. Concerning the growth feature of *B. paramycoides* 24522 (Supplementary Figure S2), it could be perceived that the strain cultured at 30°C, pH 6, and 200 rpm for 24 h can be used as the optimal inoculum for subsequent SSRE optimization.

#### 3.2.2 SSRE optimization

The ideal temperature, pH, and rotation speed for Se NPs production by microorganisms differ from species to species (Ikram et al., 2021; Romano et al., 2022). Herein, one-factor approach experiments were applied to explore these conditions on the SSRE of *B. paramycoides* 24522. The maximum SSRE was perceived at 37°C, recording 40.61%, as displayed in Figure 4D. The initial pH value affects the chargeability and permeability of the bacterial cell membrane and has an essential influence on the growth of the bacteria and the oxidation potential of Na_2_SeO_3_, which further affects the rate of Na_2_SeO_3_ reduction. The optimal pH for SSRE of *B. paramycoides* 24522 was observed at pH 6 (Figure 4E), which was similar to *Lactobacillus paracasei* HM1 (El-Saadony et al., 2021). Rotation speed is one of the critical factors affecting the dissolved oxygen level of the medium during cultivation (You et al., 2021). Therefore, it was speculated that rotation speed might be one of the crucial factors affecting the SSRE of *B. paramycoides* 24522, which is a strictly aerobic strain. As demonstrated in Figure 4F, the SSRE was gradually augmented with increasing rotation speed, and the maximum SSRE (99.39%) was achieved at 150 rpm but decreased significantly when the rotation speed was between 200 and 300 rpm.

Based on the results of one-factor optimization, BBD was used to optimize the conditions for SSRE of *B. paramycoides* 24522. The results are displayed in Table 1. Quadratic equations indicating the linear relationship between response (SSRE) and independent variables [pH (A), temperature (B), and rotation speed (C)] were:

$$Y=97.20+1.35A-5.56B-5.83C-2.22AB+1.55AC-6.56BC-21.73A^{2}-31.52B^{2}-6.70C^{2}$$

**TABLE 1:** Experimental design with the corresponding values observed by studying the combined effect of pH (A), temperature (B) and rotation speed (C) on the SSRE of *B. paramycoides* 24522.

Design points	pH (A)	Temperature (B)/^o^C	Rotation speed (C)/(r/min)	SSRE (Y)/%
1	5	35	150	40.35
2	7	35	150	57.22
3	5	40	150	35.15
4	7	40	150	43.13
5	5	37.5	120	80.26
6	7	37.5	120	70.14
7	5	37.5	180	64.32
8	7	37.5	180	60.38
9	6	35	120	63.96
10	6	40	120	64.48
11	6	35	180	66.61
12	6	40	180	40.89
13	6	37.5	150	99.64
14	6	37.5	150	95.1
15	6	37.5	150	98.27
16	6	37.5	150	97.63
17	6	37.5	150	95.38

The ANOVA of SSRE is shown in Table 2. The *p*-value of the model was 0.0001, meaning high significance (95% confidence) and indicating that the built quadratic equation was relatively credible for evaluation of the Na_2_SeO_3_ reducing ability of *B. paramycoides* 24522. In addition, all the coefficients of the equation to determine SSRE were significant (p≦0.05), except pH (A, *p* = 0.5100) and the combined effect of temperature and rotation speed (AC, *p* = 0.5915). The quadratic effects of both pH and temperature (*p* < 0.0001) were very significant.

**TABLE 2:** ANOVA of the Quadratic model for response: SSRE.

Source	Sum of squares	df	Mean square	F-value	*p*-value	Significant
Model	7621.56	9	846.84	28.03	<0.01	**
A-pH	14.55	1	14.55	0.4818	0.510	—
B- Temperature	247.42	1	247.42	8.19	0.024	*
C-Rotation speed	271.91	1	271.91	9.00	0.020	*
AB	19.76	1	19.76	0.6541	0.445	*
AC	9.55	1	9.55	0.3161	0.591	
BC	172.13	1	172.13	5.70	0.048	*
A^2^	1987.40	1	1987.40	65.79	<0.01	**
B^2^	4182.07	1	4182.07	138.44	<0.01	**
C^2^	189.19	1	189.19	6.26	0.040	*
Residual	211.45	7	30.21	—	—	—
Lack of Fit	196.45	3	65.48	17.46	<0.01	**
Pure Error	15.01	4	3.75	—	—	—
Cor Total	7833.01	16	—	—	—	—

Note: * means significant, ** means very significant.

**FIGURE 5:**
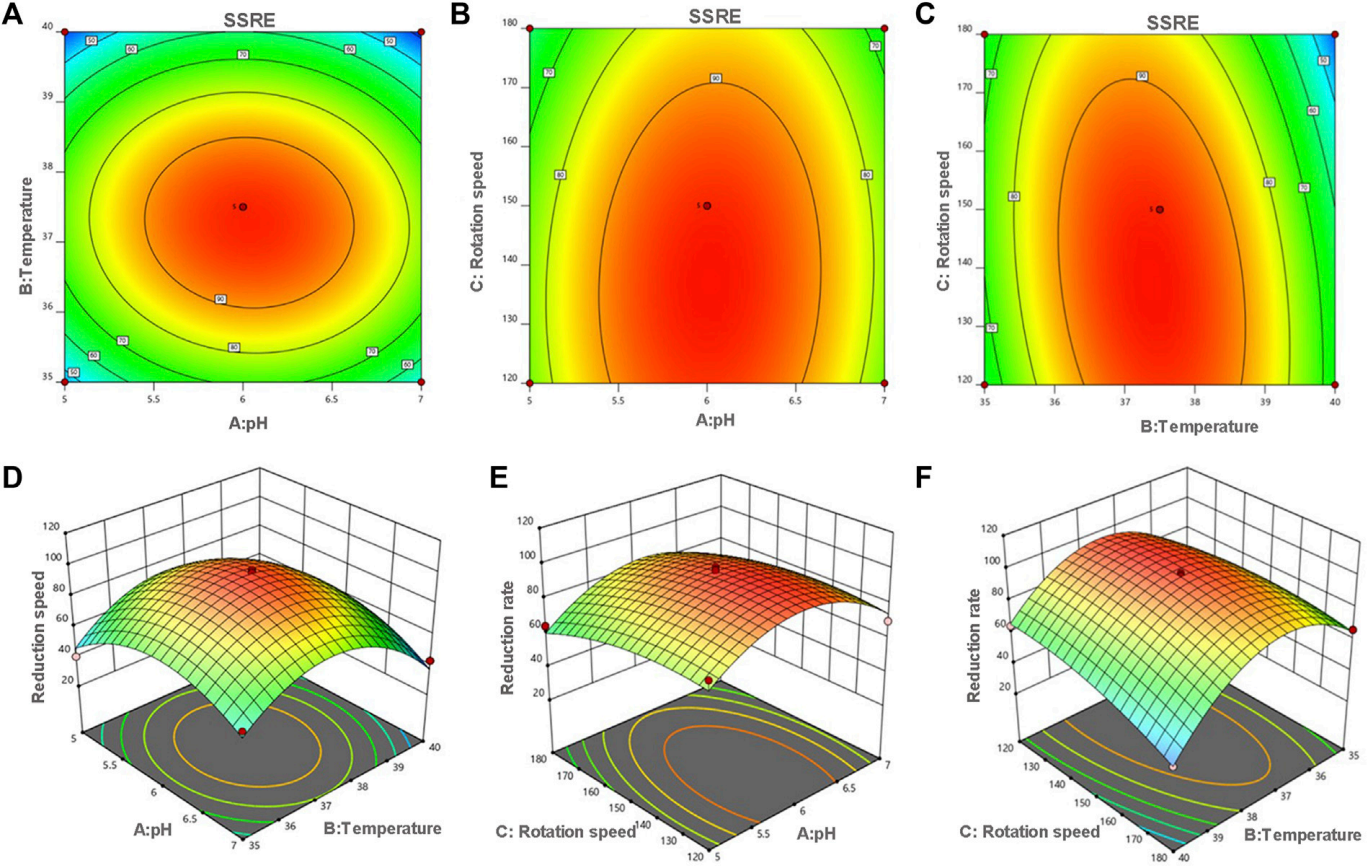
Contour **(A–C)** and response surface plots **(D–F)** of the reduction rate as a function of pH, temperature and rotation speed. The flags indicate the optimum reduction rate predicted values by the models. Red points indicate the design points above the model. Pink points indicate the design points below the model.

The optimum pH, temperature, and rotation speed for SSRE of *B. paramycoides* 24522 were 6.018, 37.383°C and 137.681 rpm, respectively. Under these conditions, the predicted responses were 98.541% (Figure 5). Considering the operational feasibility, the optimal conditions for SSRE of *B. paramycoides* 24522 were set to pH 6, 37°C, and 140 r/min. Under optimal conditions, we conducted experiments and found that the SSRE was 99.12%, which was in line with the prediction (98.541%). This indicated that the simulation model could predict the relationship between each factor and SSRE well.

### 3.3 Characterization of SeNPs synthesized by *B. paramycoides* 24522

**FIGURE 6:**
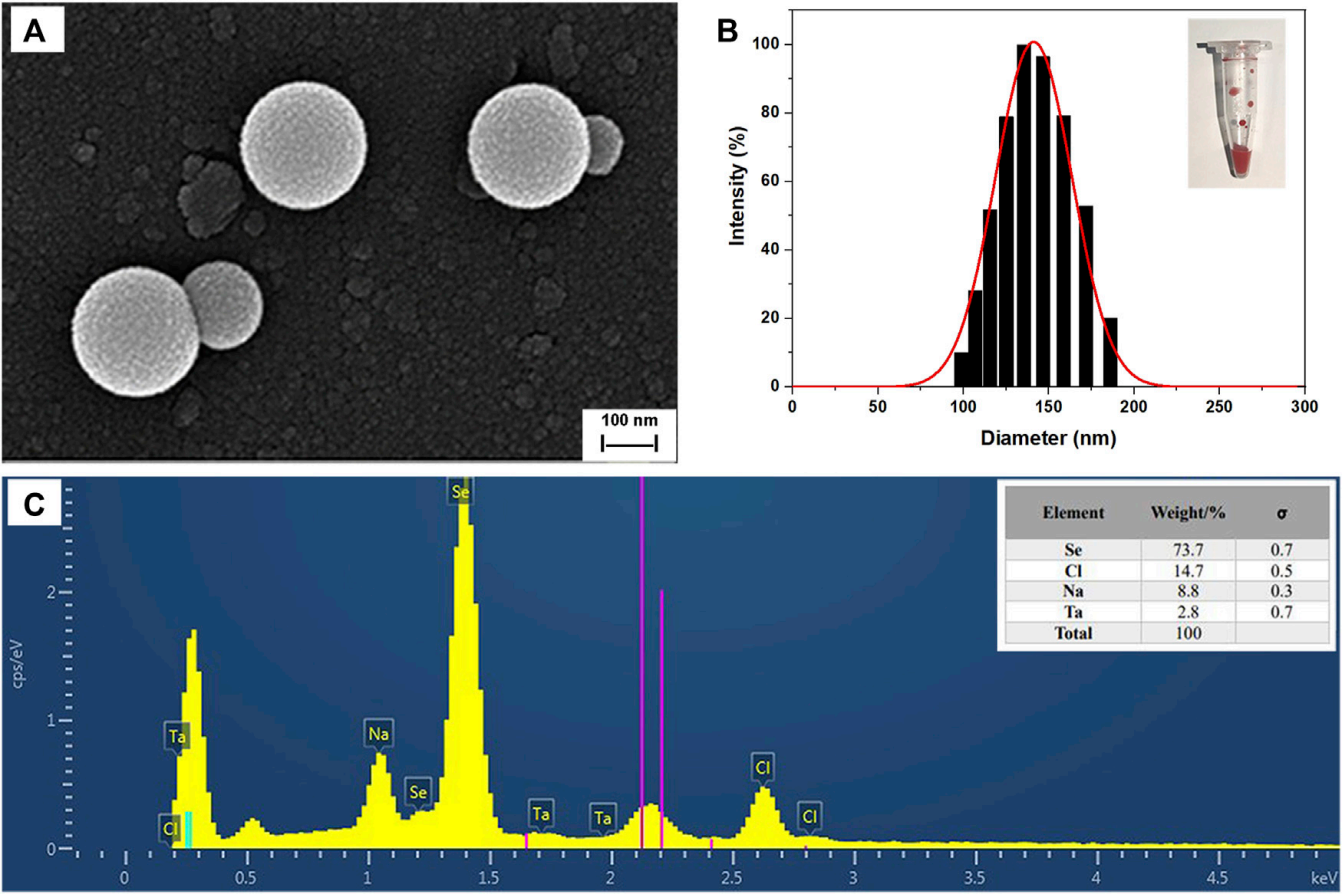
Characterization of SeNPs synthesized by *B. paramycoides* 24522. **(A)** SEM image and **(B)** size distribution histograms of SeNPs. The insets show the corresponding photographs of SeNPs. **(C)** EDS analysis of the contents of selenium (Se), chlorine (Cl), and sodium (Na) in the area of **(A)**.

SEM images of SeNPs synthesized by *B. paramycoides* 24522 with 2 mM Na_2_SeO_3_-supplemented LB broth at 24 h are given in Figure 6A. The as-prepared SeNPs were well dispersed with fairly regular spherical shapes. The particle size varied from 100 to 180 nm, and the mean size was ∼150 nm in the DLS analysis (Figure 6B). The EDS spectra (Figure 6C) displayed a strong signal in the Se area, indicating the presence of Se in the SeNPs with a weight of 73.7% (insert of Figure 6C). The SeNPs displayed an absorption peak at ∼1.4 keV, which was consistent with previous reports (Xu et al., 2018).

**FIGURE 7:**
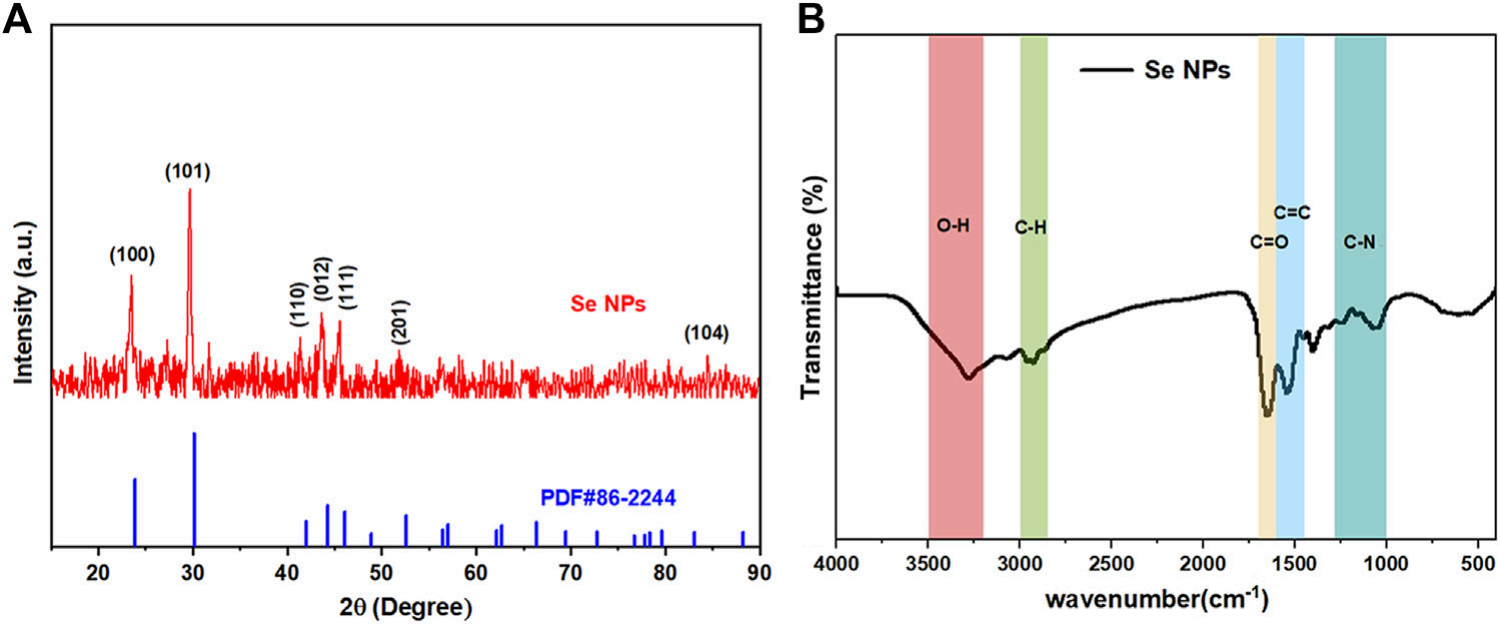
Crystallinity, surface bonding, and functional group analysis of the biosynthesized Se NPs. **(A)** XRD spectrum of the prepared Se NPs; **(B)** FT-IR spectrum of the prepared Se NPs.

The crystal composition and average crystal size of biosynthesized Se NPs were analyzed using XRD, as it provides information on the state of observed atoms. XRD studies, shown in Figure 7A, were conducted on Se NPs synthesized by *B. paramycoides* 24522. The results indicated that the Se NPs had a mix of crystalline and amorphous compositions (Velayati et al., 2022). Additionally, the XRD analysis of the biosynthesized Se NPs, shown in Figure 7A, revealed diffraction peaks at 2ɵ values of 23.875°, 30.140°, 41.998°, 44.281°, 46.08°, 52.549° and 83.084°, corresponding to Bragg’s reflections at (100), (101), (110), (012), (111), (201), and (104), respectively.

Figure 7B displays the FT-IR spectrum of Se NPs synthesized using *B. paramycoides* 24522. The spectrum reveals five clear peaks at 3274.34, 2928.78, 1653.31, 1543.26, and 1042.90. The band at 3274.34 cm^-1^ indicates the–O– vibrations of aliphatic amines. The IR bands at 2928.78 and 1,543.26 cm^−1^ correspond to the characteristics of C–H and C=O stretching, respectively. Additionally, the peaks at 1,653.31 cm^−1^ and 1,042.90 cm^−1^ suggest the presence of C=O and N–H groups, including primary and secondary amines and amides (Fan et al., 2020). These findings suggest that the protein molecule functions as a stabilizing agent to prevent and reduce the presence of agents in forming Se NPs, thereby suggesting that the synthesized protein is a key component of the final product.

### 3.4 Antibacterial and antioxidant activity of Se NPs

#### 3.4.1 Antibacterial activity

The Se NPs showed a significant inhibitory effect against *S. aureus* and *E. coli*, with inhibition zones of 16.01 ± 0.51 mm and 14.12 ± 0.89 mm, respectively. In comparison, the Na_2_SeO_3_ solution (2 mM) exhibited inhibition zones of 15.75 ± 0.77 mm and 8.04 ± 1.01 mm for *S. aureus* and *E. coli,* respectively. These findings suggest that Se NPs are more effective at inhibiting bacterial growth than Na_2_SeO_3_ solution. The *B. paramycoides* 24522 culture, however, did not show any zone of inhibition for either *S. aureus* or *E. coli*. Additionally, *S. aureus* was inhibited (MIC) at 400 μg/mL and killed (MBC) at 600 μg/mL, while *E. coli* was inhibited at 600 μg/mL and killed at 800 μg/mL (Table 3). These results demonstrate the potential of Se NPs as antibacterial agents, and further research is necessary to explore their broader applications in healthcare and environmental settings.

**TABLE 3:** Antibacterial capability of the Se NPs against pathogens.

Tested bacterial strains			Zone of inhibition (mm)									MIC and MBCs of the bio-synthesized Se NPs																	
			The bio-synthesized Se NPs			Na_2_SeO_3_ solution			*B. paramycoides* 24522			Concentrations (μg/mL)																	
												100			200			400			600			800			1000		
*S. aureus*			16.01 ± 0.51*			15.75 ± 0.77**			0			**+++**			++			×			o			NG			NG		
*E. coli*			13.26 ± 1.12**			0			0			**+++**			+++			++			**×**			o			NG		

+++Extreme growth; ++: Moderate growth; ×: MIC; o: MBC; NG: No growth. ** represents slightly significant and * represents a non-significant difference from control at *p* < 0.05 by one-way ANOVA, in the column Values are mean ± SD, of triplicate.

#### 3.4.2 Antioxidant activity

**FIGURE 8:**
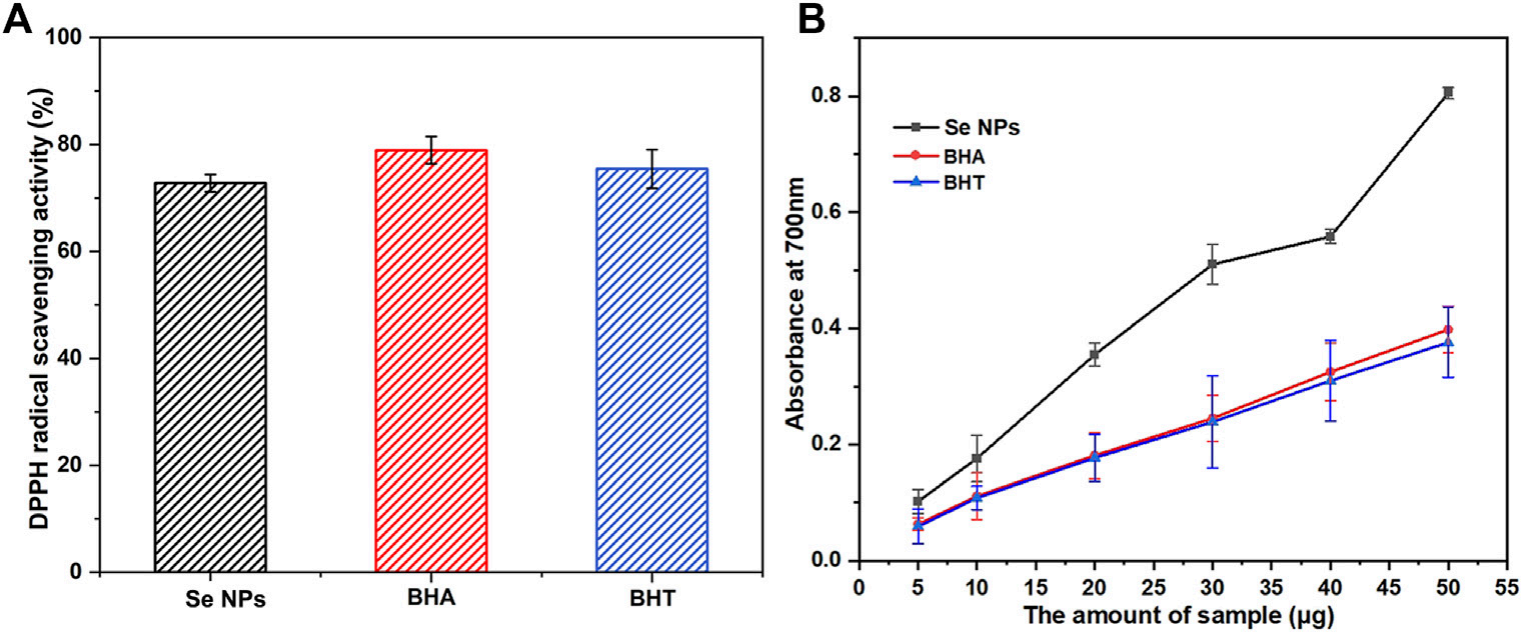
The scavenging ability on 1,1-diphenyl-2-picrylhydrazil (DPPH) radicals **(A)** and reducing capacity **(B)** of the bio-synthesized Se NPs, butyl hydroxy anisole (BHA) and butylated hydroxytoluene (BHT).

This study also investigated the potential antioxidant activity of biosynthesized Se NPs compared to the synthetic antioxidants BHA and BHT. As shown in Figure 8A, the Se NPs showed a DPPH radical scavenging activity of 72.79% at a concentration of 100 μg/mL, which was comparable with the standards, BHA and BHT. This finding is significant because it suggests that Se NPs may have potential applications in the food and pharmaceutical industries as antioxidants. The reducing capacity of all tested samples increased with increasing amounts, and the Se NPs showed the highest reducing capacity, followed by BHA and BHT (Figure 8B). This result was consistent with the DPPH free radical scavenging activity, which also demonstrated that the biosynthesized Se NPs had strong antioxidant activity. These findings have important implications for the development of eco-friendly, sustainable and natural antioxidants.

## 4 Discussion

The aim of the present study was to synthesize Se NPs using the biogenic selenate-reducing bacterium *B. paramycoides* 24522 and investigate their antibacterial and antioxidant activities. Our results showed that *B. paramycoides* 24522 efficiently reduced 2 mM Na_2_SeO_3_ with a maximal SSRE of 99.12% at 37°C, pH 6, and 140 r/min within 24 h. The synthesized Se NPs exhibited a stable red color and demonstrated significant antibacterial activity against *S. aureus* and *E. coli*, with MICs ranging from 400 μg/mL to 600 μg/mL and MBCs ranging from 600 μg/mL to 800 μg/mL. Additionally, the Se NPs showed promising antioxidant capabilities through scavenging DPPH radicals and reducing capacity.

Our findings suggest that that protein molecules function as a stabilizing agent in the biosynthesis of Se NPs by *B. paramycoides* 24522. Furthermore, five different proteins exocytosis protein (ECP), periplasmic space protein (PSP), cytoplasmic protein (CPP), cell membrane and cell wall protein (MWP), and intracellular polysaccharides (IPS), were isolated from the bacteria using ultrasonic fragmentation (Supplementary Figure S3). And CPP from the cytoplasmic protein played a pivotal role in the synthesis of Se NPs. FT-IR spectroscopy results also demonstrated the critical role of proteins in facilitating the biosynthesis of Se NPs, providing insights into the mechanisms involved in synthesizing these nanoparticles through biological means. However, which protein plays the crucial role in sodium selenite reduction and Se NPs synthesis from MWP remains to be investigated. The research also highlights the potential utility of Se NPs as an environmentally friendly solution to selenium pollution and in various applications such as new drug delivery systems, sensors, and photocatalysts.

Compared to previous studies, our optimized biosynthesis method taking only 24 h is time-efficient (Table 4). For instance, Kora et al. (Kora and Rastogi, 2016; Kora, 2018) found that both *Pseudomonas aeruginosa* ATCC 27853 and *Bacillus cereus* AJ3 take 24–72 h to prepare SeNPs; Khoei et al. (Khoei et al., 2017) and Song et al. (Song et al., 2017) reported that *Burkholderia fungorum* DBT1, *Burkholderia fungorum* 95, and *Enterobacter cloacae* Z0206 take 96 h to complete Se NPs synthesis. Meanwhile, *B. paramycoides*, isolated in this work, is a highly beneficial bacteria that can effectively break down organic matter and remove pollutants, and has great potential for improving environmental sustainability and reducing the harmful impact of waste on our planet.

**TABLE 4:** Various conditions for the green synthesis of SeNPs by different bacteria.

Bacteria	Operating condition (concentration of precursor, temperature, pH, etc.)	Duration for synthesis/h	Size (nm)	Reference
*Pseudomonas aeruginosa* ATCC 27853	0.25–1 mM Na_2_SeO_3_, at 37°C under static conditions	24–72	95.9	Kora and Rastogi (2016)
*Burkholderia fungorum* DBT1 and *Burkholderia fungorum* 95	0.5 mM and 1 mM of Na_2_SeO_3_, at 27°C	96	170 and 200	Khoei et al. (2017)
*Enterobacter cloacae* Z0206	0.5–15 mM Na_2_SeO_3_, at 32°C, 250 rpm	96	100–300	Song et al. (2017)
*Lactobacillus casei*	1.2 mM Na_2_SeO_3_, at 37°C under anaerobic conditions	24	50–80	Xu et al. (2018)
ATCC 393				
*Bacillus cereus* AJ3	1 mM Na_2_SeO_3_, at 37°C	24–72	93	Kora (2018)
*Stenotrophomonas maltophilia* SeITE02	0.5 mM Na_2_SeO_3_, at 27°C, 200 rpm	48	160–250	(Cremonini et al., 2018)
*Mariannaea* sp. HJ	2 mM SeO_2,_ pH 10, aerobically cultivated at 30°C	32	45.19^a^, 12.65^b^	Zhang et al. (2019)
*Saccharomyces cerevisiae*	5 µg Na_2_SeO_3_, at 32°C, 120 rpm	96	50	Faramarzi et al. (2020)
*Lactobacillus paracasei* HM1	4.0 mM Na_2_SeO_3_, at 35°C, pH 6, 160 rpm	32	56.91	El-Saadony et al. (2021)
*Bacillus paramycoides* SP3	10 mM Na_2_SeO_3_, at 30°C, 150 rpm	72	149	Borah et al. (2021)
*Fusarium oxysporum*	2 mM SeCl_4_, at room temperature, 200 rpm	72	42	Islam et al. (2022)
*Bacillus paramycoides* 24522	2 mM Na_2_SeO_3_, at 37°C, 140 rpm, pH 6	24	150	This work

^a^ Size of intracellular SeNPs.

^b^ Size of extracellular SeNPs.

It is noteworthy that Borah et al. (Borah et al., 2021) reported that *B. paramycoides* SP3, which was isolated from the leachate of coal mine overburden, is capable of synthesizing Se NPs within 72 h. However, our isolate, *B. paramycoides* 24522, differs in two aspects from the aforementioned isolate: (1) it was obtained from sediments of Se-enriched culture ponds, and (2) it can accomplish synthesis within 24 h with a remarkable sodium selenite reduction efficiency of 99.12%.

Moreover, the biogenic Se NPs have been recently discovered to possess an exceptional ability to combat bacterial infections, making them a highly promising and potent agent in the field of antibacterial research. Studies have shown that Se NPs have broad-spectrum antibacterial effects that target both gram-positive and gram-negative bacteria (Indhira et al., 2023). The antibacterial activity of Se NPs is attributed to several mechanisms, including disintegration of the bacterial cell wall and membrane, hampering bacterial metabolism and impeding bacterial DNA replication (Abadi et al., 2022). When compared to traditional antibiotics, Se NPs have been shown to have advantages such as a smaller size, higher surface area-to-volume ratio, and greater ability to penetrate bacterial cell walls, which make them effective antimicrobial agents. In addition, while the antimicrobial activity of Se NPs may not be as strong as that of Ag NPs against certain microorganisms (Davoodbasha et al., 2016), they have been shown to be effective against a wider range of microorganisms. Therefore, Se NPs may be a promising alternative to traditional antibiotics and other antimicrobial agents. Se NPs also possess potent antioxidant activity, which can effectively scavenge free radicals and reactive oxygen species (ROS) in cells, thereby protecting against diseases such as cancer, diabetes, and neurodegenerative disorders (Hashem et al., 2023). Due to their outstanding antioxidant properties, Se NPs have been identified as promising therapeutic candidates for treating oxidative stress-related diseases (Fan et al., 2020).

In conclusion, the present study demonstrates the efficient biosynthesis of Se NPs using *B. paramycoides* 24522 and their potential applications in addressing selenium pollution, as well as in the fields of environment and biotechnology. The synthesized Se NPs exhibited significant antibacterial and antioxidant activities, indicating their potential use as antibacterial agents and therapeutic candidates for treating oxidative stress-related diseases. Further research is needed to identify the specific protein responsible for Se NP synthesis and to investigate their potential toxicity and efficacy *in vivo*.

## Data Availability

The original contributions presented in the study are included in the article/Supplementary Material, further inquiries can be directed to the corresponding author.
