# Supplementary material for: Highly-efficient synthesis of biogenic selenium nanoparticles by Bacillus paramycoides and their antibacterial and antioxidant activities
Source: Front Bioeng Biotechnol. 2023 Aug 1;11:1227619. doi: 10.3389/fbioe.2023.1227619 (PMC10429174; doi:10.3389/fbioe.2023.1227619)
Supplement: Supplementary file 1 [file DataSheet1.docx]

Supplementary Material

Highly-efficient Synthesis of Biogenic Selenium Nanoparticles by *Bacillus paramycoides* and their Antibacterial and Antioxidant Activities

Pei Liu^1,2ǂ^, Haiyu Long^1,2ǂ^, Han Cheng^1,2^, Mengdi Liang^1,2^, Zhengwei Liu^1,2^, Zhenlian Han^3^, Zhen Guo^1,2^, Hao Shi^1,2^, Min Sun^4^, Shuai He^1,2*^

*** Correspondence:** Shuai He, [heshuai@hyit.edu.cn](mailto:heshuai@hyit.edu.cn)

# Supplementary Figures and Tables

## Supplementary Figures





**Supplementary Figure 1.** The standard curve of elemental selenium quality





**Supplementary Figure 2.** Growth curve of strain 24522

**
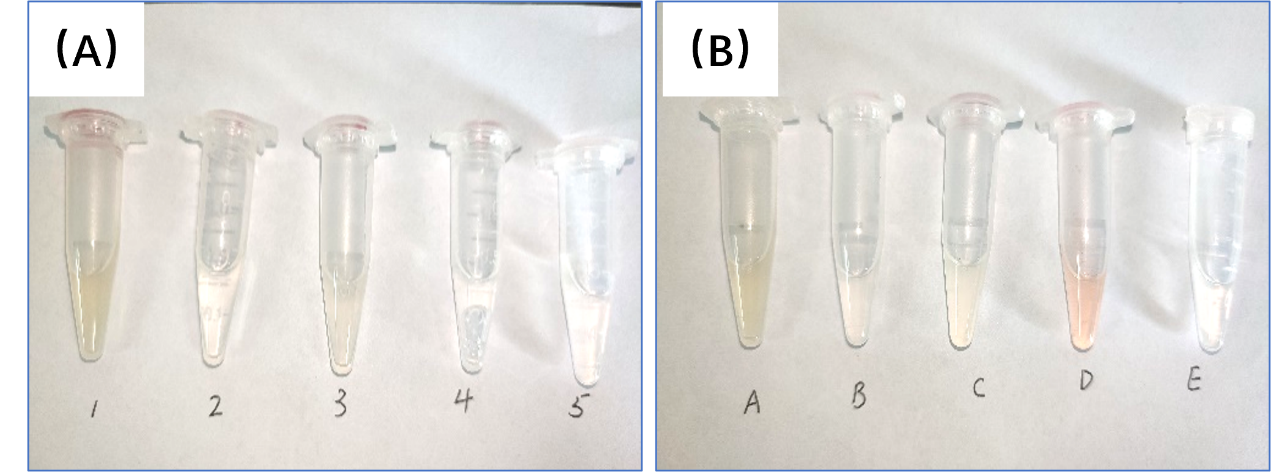
**

**Supplementary Figure 3.** Verification of the ability of five different proteins: exocytosis protein (ECP), periplasmic space protein (PSP), cell membrane and cell wall protein (MWP), cytoplasmic protein (CPP), and Intracellular polysaccharides (IPS) and polysaccharides to reduce Na_2_SeO_3_. (A) represents the control experiments without Na_2_SeO_3_, (B) represents the sample experiments with Na_2_SeO_3_.

**
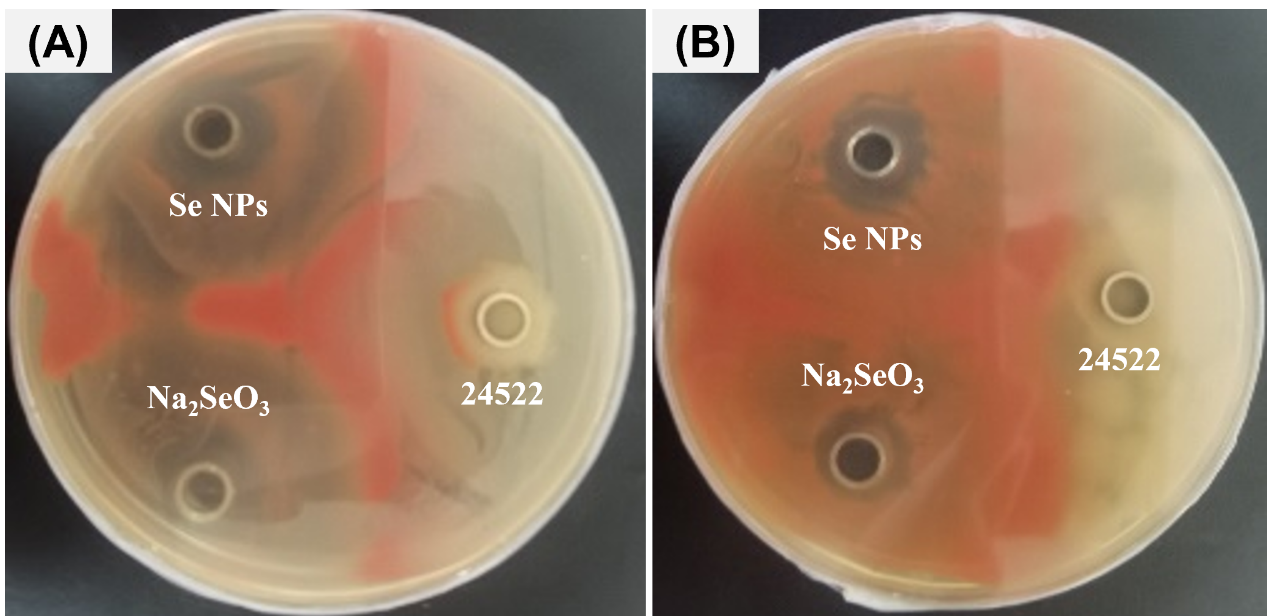
**

**Supplementary Figure 4.** Antimicrobial activity of Se NPs, Na_2_SeO_3_ solution and *B. paramycoides* 24522 against t *S. aureus* (A) and *E. coli* (B).

## Supplementary Tables

**Supplementary Table 1.** The physiology and biochemistry of strain 1805

| Characteristic | | | Growth status of strain 1805 |
| --- | --- | --- | --- |
| Temperature | 20℃, 25℃, 30℃, 37℃, 40℃, 42℃ | + | |
|  | 4℃, 50℃ | - | |
| pH | 4, 5, 6, 7, 8, 9, 10, 11 | + | |
|  | 3,12 | - | |
| Gram stain test | | + | |
| Catalase test | | + | |
| V-P test | | + | |
| Casein test | | + | |
| Starch hydrolysis test | | + | |

+ represents strain 1805 gorws well, - represents strain 1805 does not gorw.

**Supplementary Table 2.** Factors and levels in response surface design

| Levels | pH | Temperature | Rotation rate |
| --- | --- | --- | --- |
|  |  | ℃ | r/min |
| -1 | 5 | 35 | 120 |
| 0 | 6 | 37 | 150 |
| 1 | 7 | 40 | 180 |
